# The effects of myelin on macrophage activation are phenotypic specific via cPLA_2_ in the context of spinal cord injury inflammation

**DOI:** 10.1038/s41598-021-85863-6

**Published:** 2021-03-18

**Authors:** Timothy J. Kopper, Bei Zhang, William M. Bailey, Kara E. Bethel, John C. Gensel

**Affiliations:** Department of Physiology, Spinal Cord and Brain Injury Research Center, University of Kentucky College of Medicine, Lexington, KY 40536 USA

**Keywords:** Neuroscience, Diseases of the nervous system, Myelin biology and repair, Neuroimmunology

## Abstract

Spinal cord injury (SCI) produces chronic, pro-inflammatory macrophage activation that impairs recovery. The mechanisms driving this chronic inflammation are not well understood. Here, we detail the effects of myelin debris on macrophage physiology and demonstrate a novel, activation state-dependent role for cytosolic phospholipase-A2 (cPLA_2_) in myelin-mediated potentiation of pro-inflammatory macrophage activation. We hypothesized that cPLA_2_ and myelin debris are key mediators of persistent pro-inflammatory macrophage responses after SCI. To test this, we examined spinal cord tissue 28-days after thoracic contusion SCI in 3-month-old female mice and observed both cPLA_2_ activation and intracellular accumulation of lipid-rich myelin debris in macrophages. In vitro*,* we utilized bone marrow-derived macrophages to determine myelin’s effects across a spectrum of activation states. We observed phenotype-specific responses with myelin potentiating only pro-inflammatory (LPS + INF-γ; M1) macrophage activation, whereas myelin did not induce pro-inflammatory responses in unstimulated or anti-inflammatory (IL-4; M2) macrophages. Specifically, myelin increased levels of pro-inflammatory cytokines, reactive oxygen species, and nitric oxide production in M1 macrophages as well as M1-mediated neurotoxicity. PACOCF3 (cPLA_2_ inhibitor) blocked myelin’s detrimental effects. Collectively, we provide novel spatiotemporal evidence that myelin and cPLA_2_ play an important role in the pathophysiology of SCI inflammation and the phenotype-specific response to myelin implicate diverse roles of myelin in neuroinflammatory conditions.

## Introduction

Spinal cord injury (SCI) triggers a complex neuroinflammatory response that mediates tissue repair but also potentiates secondary injury processes. Activated macrophages, consisting of resident microglia and infiltrating monocytes, contribute to this dichotomous response. Macrophages facilitate repair by increasing axon growth, stem cell differentiation, and revascularization^1,2^, however, macrophages can also contribute to pathology through secondary injury processes involving reactive oxygen species (ROS), neurotoxins, and pro-inflammatory cytokine release as well as by causing axon retraction and dieback^1,3^. The extent to which macrophages are polarized toward reparative (also called M2 or anti-inflammatory) or pathological (also called M1 or pro-inflammatory) phenotypes largely depends on the stimuli present in the injured spinal cord.

One notable distinction between SCI and self-resolving peripheral nerve injuries is the sustained presence of myelin debris. Lipid-laden myelin debris is taken up and processed predominantly by infiltrating macrophages, rather than endogenous microglia, after SCI^4^. Phagocytic markers are present on these chronically activated macrophages, which become visibly laden with debris^4–8^. It is therefore likely that myelin lipids are actively processed by macrophages and are environmental stimuli that drive chronic spinal cord inflammation. Indeed, in areas of Wallerian degeneration, macrophages disappear from the chronically injured spinal cord concomitant with myelin debris clearance^4–6,9^. The accumulation of lipid debris in the days and weeks after injury also closely aligns with the temporal shift in macrophage phenotype, ending with persistent pro-inflammatory activation by 28 dpi^2,4^. Evidence also suggests that myelin acts as an inflammatory stimulus on macrophages in vitro, implicating a key link between myelin debris accumulation and the grievous shift in macrophage phenotype which impairs regeneration after SCI^4,7,10,11^.

While myelin debris is implicated in macrophage activation state and recovery after SCI^4,7,12^, the intracellular mechanisms mediating myelin’s effects remain unclear. Cytosolic phospholipase A2 (cPLA_2_) facilitates arachidonic acid (AA) release from cellular membranes following inflammatory stimuli and is largely unstudied in SCI macrophage responses. Interestingly, myelin membranes contain high concentrations of AA, stored in its inactive esterified state; however, whether cPLA_2_ can act on these lipids remains unknown. cPLA_2_-mediated breakdown of AA initiates an eicosanoid storm in which a wide variety of bioactive lipids are released including prostaglandins, leukotrienes, and thromboxanes. Eicosanoids have diverse albeit largely pro-inflammatory functions including activating the inflammatory NF-kB signaling cascade and increasing edema as well as potentiating immune cell chemoattraction, fibrosis, and inflammatory responses. The role of cPLA_2_ in macrophage physiology has been detailed in other systems; however, it is unknown if cPLA_2_ has any differential effects in macrophages polarized along the spectrum of activation states in the presence of myelin, or if it is a contributor to prolonged pro-inflammatory activation of macrophages after SCI. cPLA_2_ activation is induced by inflammatory stimuli such as LPS/IFN-γ, also known as the M1, pro-inflammatory paradigm in vitro*,* and is likely induced by the complex inflammatory environment observed after SCI^13–15^. This is the basis of our hypothesis that cPLA_2_ activity in myelin-laden macrophages after SCI aggravates tissue damage and contributes to chronic inflammation. Here we establish these mechanisms in vitro, and provide the proof-of-concept that these pathways may play an important role in vivo after SCI.

## Methods

### Animals

As described previously^16,17^, in vitro experiments were performed using 2–4-month-old female C57BL/6 mice (Jackson Laboratory, Bar Harbor, Maine). In vivo experiments were performed using 4-month-old female C57BL/6 mice, weighing 20.7 g ± 1.3 g (Jackson Laboratory, Bar Harbor, ME, USA). Animals were housed in IVC cages with ad libitum access to food and water. All procedures were performed in accordance with the guidelines and protocols of the Office of Research Integrity and with approval of the Institutional Animal Care and Use Committee at the University of Kentucky. All experiments were carried out in compliance with the ARRIVE guidelines^18^.

### Cell culture

Bone marrow-derived macrophages (BMDMs) were extracted from the femur and tibia of female C57BL/6 mice at 2–4 months old as previously reported^19,20^ and were plated at 0.8–1 × 10^6^ cells/mL in differentiation media containing Roswell Park Memorial Institute medium (RPMI, Thermo Fisher Scientific, #21870-092) supplemented with 1% penicillin/streptomycin (P/S, Thermo Fisher Scientific, #5140122), 1% HEPES (Sigma-Aldrich, #83264-100ML-F), 1% GlutaMAX 0.001 (Thermo Fisher Scientific, #35050061) 0.001% β-mercaptoethanol (Thermo Fisher Scientific, #21985023), 10% FBS (Life technologies, #10082147), and 20% supernatant from sL929 cells (a generous gift from Phillip Popovich, The Ohio State University). Supernatant collected from sL929 cells contains macrophage colony-stimulating factor, which helps to promote bone marrow cells’ differentiation into macrophages^21^. The BMDMs were allowed to differentiate for 7 days in culture, and cells were then replated on day 7 at a density of 1 × 10^6^ cells/mL in 12-well plates in RPMI, containing 1% P/S, 1% GlutaMAX and 10% FBS. On day 8, cells were stimulated for 24 h to be either M1 using LPS (50 ng/mL, Invivogen, #tlrl-eblps, standard preparation) plus IFN-γ (20 ng/ml, eBioscience #14-8311-63) diluted in N2A growth medium (described below), M2 using IL-4 (20 ng/ml, R&D systems, #404-ML-010), or Control/Unstimulated (CTL) using fresh media without any stimulants. At the time of stimulation cells were immediately treated with myelin debris (50 µL/mL, preparation described below), 50 µM PACOCF3 (inhibitor of cPLA_2_, Tocris Bioscience, CAS 141022-99-3), or PBS/DMSO vehicles to equalize volume and drug solvent concentrations across groups. 24 h after stimulation the supernatants were removed, centrifuged at 13,000 RPM (Fisher Scientific accuSpin Micro R centrifuge), and then this macrophage conditioned media (MCM) was either applied directly to N2A cells to measure cytotoxicity, or stored at − 80 °C prior to testing for IL-12p40 levels using standard ELISA kits (Thermo Fisher Scientific, Rockford, IL # EMIL12P40), Nitric Oxide with the Griess Reagent Kit (Thermo Fisher Scientific # G-7921), and phenol red-free RPMI, or a mulit-plex ELISA system measuring protein levels of TNF-alpha, IL-1Beta, IL-6, CX3CL1, and IL-10 (Meso Scale Diagnostics). BMDMs for coverslip stains were treated as above, except at a lower plating density of 3 × 10^5^ cells/mL. Coverslips were fixed in cold 2% PFA for 30 min, washed in PBS and stored at 4 °C until staining.

Moderate purity myelin (> 95% myelin, with small contributions from axolemma and other cellular membranes) was prepared as follows (adapted from Larocca et al.^22^): brains were collected from C57BL/6 mice at the time of BMDM isolation and stored at − 80 °C prior to myelin isolation. The brains were rinsed and suspended in cold PBS with 1% P/S and placed in a Dounce homogenizer (DWK Life Science, #357544) under sterile conditions and blended with the loose and tight pestles. The solution was transferred to a 15 mL tube and pelleted at 2000 RPM (Thermo Scientific Legend XTR centrifuge) prior to discarding the soluble supernatant fraction. The pellet was resuspended in the PBS/P/S, and then 5mLs of a 30% Percoll solution (Sigma-Aldrich, #P1644-500ML) was gently underlaid below the myelin solution for density gradient centrifugation. The layers were then centrifuged at 2000 RPM for 15 min at 4 °C under gentle acceleration/deceleration, generating three distinct layers (soluble on top, myelin in middle, and Percoll/cell pellet on bottom). After removing the soluble fraction, the myelin was transferred to a fresh tube and resuspended in 10 mL distilled water with 1% P/S and incubated for 10 min (hypoosmotic shock) to separate membranes at 4 °C. The myelin was then re-pelleted at 2000 RPM, suspended in PBS/1% P/S and separated a second time by density gradient centrifugation as described above. The myelin was then suspended and pelleted twice in PBS/1% P/S to remove residual Percoll and water-soluble contaminants, and then aliquoted before storage at − 80 °C. The final protein concentration of the myelin stock solutions produced by this protocol were 10.23 mg/mL with a standard deviation of 0.282 mg/mL as determined by a BCA Protein Assay Kit (Thermo Fisher Scientific #23225). With the application of myelin debris to BMDMs at 50 µL/mL, cells had a mean dosage of 0.51 mg/mL. Lastly, to ensure our results were not due to endotoxin contamination in our myelin preparations, we tested aliquots from each batch of myelin stimulant (Thermo Fisher Scientific #88282).

A mouse neuroblastoma cell line (Neuro-2a or N2A, a gift from Chris Richards, University of Kentucky) was maintained in N2A growth medium containing 45% DMEM, 45% OPTI-MEM reduced-serum medium, 10% fetal bovine serum (FBS), and 1% penicillin/streptomycin. N2A were plated at a density of 1 × 10^5^ cells/mL in 96-well tissue culture plates and allowed to proliferate for 48 h. The neurotoxicity of MCM was evaluated as reported previously using a MTT-based cell growth determination kit according to the manufacturer’s instructions (Sigma-Aldrich CGD1-1KT)^23^. Briefly, 24 h before testing, N2A growth media was replaced with serum-free N2A media to induce differentiation. The day of testing this media was replaced by fresh MCM, and the N2A cells were incubated in MCM for 24 h before thiazolyl blue tetrazolium bromide (MTT (5 mg/ml), 20 μl per well) was added to each well and the cells further incubated for 2 h. The tetrazolium ring of MTT can be cleaved by mitochondrial dehydrogenases of viable cells, yielding purple formazan crystals, which were then dissolved in acidified isopropanol solvent. The resulting purple solution was spectro-photometrically measured at 570 nm Epoch microplate reader (BioTek Instruments, Inc., Winooski, VT) using 690 nm as a background absorbance. This data is normalized to the non-toxic CTL values to generate proportional decrease in viability values and presented inversely as increased toxicity relative to CTL.

Macrophage reactive oxygen species (ROS) production was measured using CM-H2DCFDA (Invitrogen #C6827). In short, BMDMs were cultured and stimulated as described above except in a 96 well plate (1 × 10^6^ cells/mL). Following the 24-h stimulation the supernatants were removed and replaced with a 5 µM solution of CM-H2DCFDA in phenol red-free RPMI with 1% GlutaMAX and penicillin/streptomycin and incubated at 37 °C for 25 min. ROS mediates the conversion of this compound to fluorescent DCF which was then detected by an Epoch microplate reader (BioTek instruments, Inc., Winooski, VT) at the compound’s Excitation/Emission spectra of approx. 492–495/517–527 nm.

Macrophage cPLA_2_ activity was measured using a Cytosolic Phospholipase A2 Assay Kit (Abcam #ab133090). In short, cells were cultured as described above except in six well culture dishes (1 × 10^6^ cells/mL). Cells were lysed and briefly sonicated on ice in TBS-T (0.4% Triton-X) with a protease inhibitor (Sigma-Aldrich #11836170001) before proceeding directly into the manufacturer’s protocol.

### Spinal cord injury

As described previously^16,17^, animals were anesthetized via intraperitoneal (i.p.) injections of ketamine (100 mg/kg) and xylazine (10 mg/kg). Following a T9 laminectomy, a moderate-severe thoracic SCI was produced using the Infinite Horizon (IH) injury device (75-kdyn displacement; Precision Systems and Instrumentation). Any animals receiving SCI with abnormalities in the force vs. time curve generated by the IH device were excluded from analysis. These abnormalities are indicative of bone hits or instability in the spinal cord at the time of injury and occurred < 10% of the time. After injury, muscle and skin incisions were closed using monofilament suture. After surgery, animals received one subcutaneous injection of buprenorphine-SR (1 mg/kg) and antibiotic (5 mg/kg, enroloxacin 2.27%: Norbrook Inc., Lenexa, KS) in 2 mL of saline and were housed in warming cages overnight. Animals continued to receive antibiotic subcutaneously in 1 mL saline for 5 days. Food and water intake and the incision site were monitored throughout the course of the study. Bladder expression was performed on injured mice twice daily. Mice were sacrificed at 7 and 28 days post-injury (n = 8 and 10, respectively) to generate spinal cord sections for histological analyses.

### Tissue processing and immunohistochemistry

As described previously^16,17^, mice were anesthetized and then transcardially perfused with cold PBS (0.1 M, pH 7.4), followed by perfusion with cold 4% paraformaldehyde (PFA). Dissected spinal cords (1 cm) were post-fixed for another 2 h in 4% PFA and subsequently rinsed and stored in cold phosphate buffer (0.2 M, pH 7.4) overnight at 4 °C. On the following day, tissues were cryoprotected in 30% sucrose for 3 days at 4 °C, followed by rapidly freezing and blocking in optimal cutting temperature (OCT) compound (SakuraFinetek USA, Inc.) on dry ice. Tissue blocks were cut in serial coronal sections (10 μm) and mounted onto Colorfrost plus slides (Fisher #12-550-17).

Spinal cord sections were stained with Eriochrome Cyanine (myelin) and anti-Neurofilament (1:1000, Aves labs, NFH) to visualize damage and thereby identify the epicenter of each lesion, as defined as the point where spared tissue constitutes the smallest proportion of spinal cord volume^16^. Immunohistochemistry on tissue sections and BMDM coverslips was performed to stain for phosphylated-cPLA_2_ (p-cPLA_2_; rabbit, 1:500, Cell Signaling Technology #2831S), BODIPY (2 µM 30 min, Thermo Fisher Scientific #D3922), biotinylated tomato lectin (TomL) (Sigma-Aldrich L0651-1MG, and DAPI (Sigma-Aldrich #D9542-10MG) overnight at 4 °C. Secondary antibodies were applied at 1:1000 for 1 h at RT: Alexa Fluor 546 goat anti-rabbit (Life Technologies #11010), and Streptavidin Alexa Fluor 647 conjugate (Thermo Fisher Scientific #S-21374). Antigen retrieval was performed to improve signal: 10 min in sodium citrate buffer (10 mM Sodium citrate, 0.05% Tween 20, pH 6.0) at 90 °C. BODIPY is specific to neutral lipids from the breakdown of myelin and cellular membranes. TomL binds to poly-N-acetyl lactosamine on macrophages and microglia. TomL also binds to large blood vessels which were excluded from analysis when possible based on their large tubular morphology^24,25^. Imaging was performed at or within 100 µm of lesion epicenter due to tissue loss during antigen retrieval. All images were taken using a C2+ laser scanning confocal microscope (Nikon Instruments Inc., Melville, NY, USA). Images were quantified using using the MetaMorph analysis program (Molecular Devices, Sunnyvale, CA, USA).

### Statistical analysis

As described previously^16,17^ statistical analyses were completed using GraphPad Prism 6.0 (GraphPad Software). Data were analyzed using one- or two-way ANOVA followed by Dunnett’s test for multiple comparisons. Results were considered statistically significant at p ≤ 0.05. All data are presented as mean ± SEM unless otherwise noted. All in vitro measurements were done in triplicates, and at least three independent experiments were carried out. Imaging and quantification were performed by investigators fully blinded to all experimental conditions. In vitro experiments were not fully blinded during experimental procedures (due to the obvious presence of myelin in some conditions); however, all analyses were confirmed by an investigator blinded to experimental conditions. Figures were prepared using Adobe Photoshop CS6 (Adobe Systems) and Prism 6.0.

## Results

### Myelin-laden macrophages contain active cPLA_2_ after SCI

**Figure 1:**
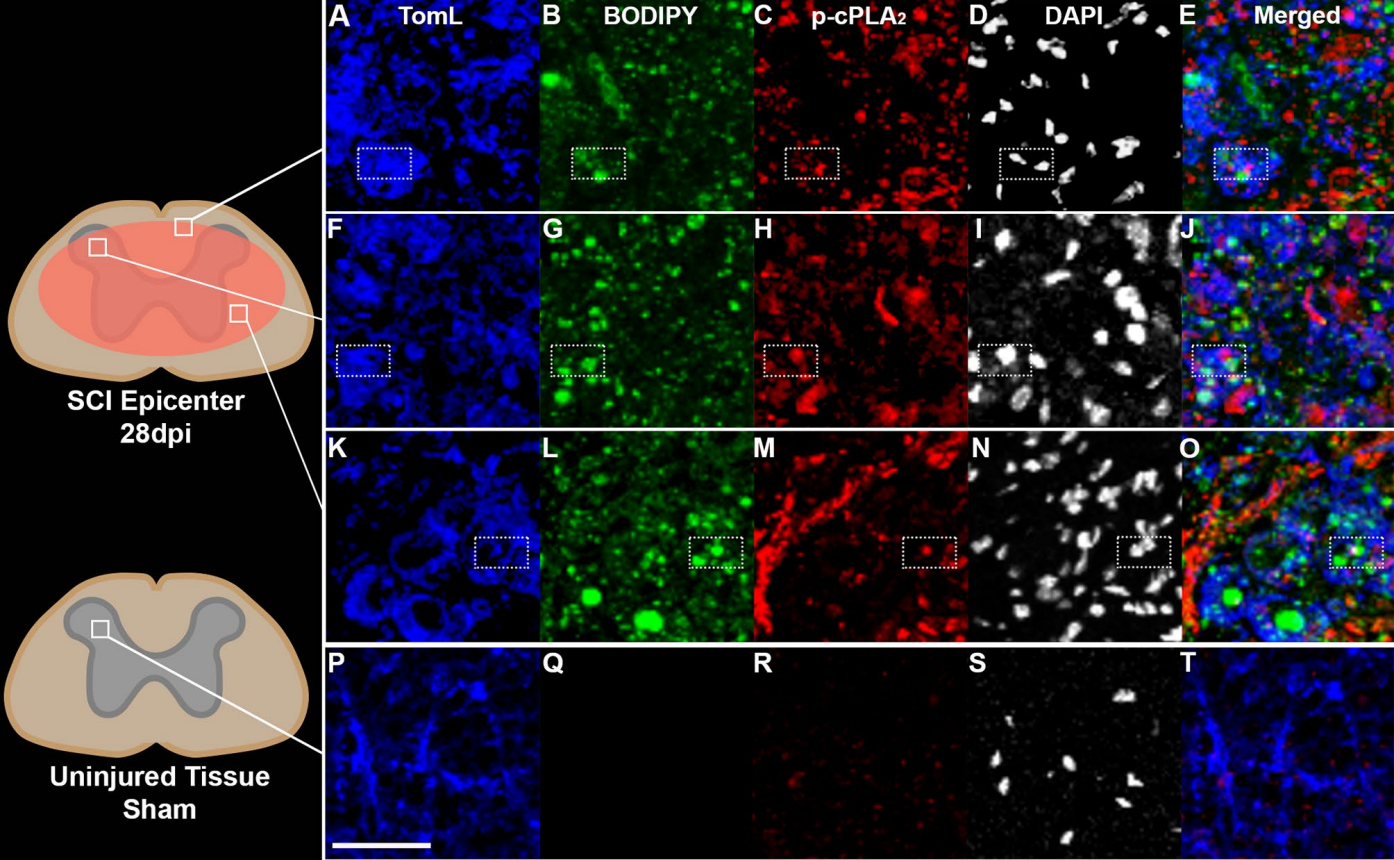
Macrophages can contain both myelin-derived lipids and active cPLA_2_ 28 days after spinal cord injury (SCI). Ten adult, 4-month-old C57/b female mice received a T9 75kdyn infinite horizons (IH) contusion SCI. (**A**–**E**) Representative example of TomL positive macrophages (blue) in injured white matter containing both lipid debris (green, BODIPY, staining for neutral lipids) and active p-cPLA_2_ (red) (imaged area is represented by box within spinal cord diagram). DAPI (white) was excluded from merged images. (**F**–**J**) Example of TomL positive macrophages (blue) in injured grey matter containing both lipid debris (green, BODIPY, staining for neutral lipids) and active p-cPLA_2_ (red). (**K**–**O**) Example of TomL positive macrophages (blue) in injured grey matter in the core of the injury epicenter containing both lipid debris (green, BODIPY, staining for neutral lipids) and active p-cPLA_2_ (red). (**P**–**T**) Example of TomL positive macrophages (blue) in uninjured tissue from sham animals with minimal lipid debris or active p-cPLA_2_ (red). Boxes indicate examples of triple positive cells. Maximum intensity projection confocal images. Scale bar in image p = 50 µm.

In order to determine the extent to which cPLA_2_ may be contributing to myelin processing by macrophages after SCI, we examined inflammation within the injured spinal cord 4 weeks after mouse contusion SCI. As reported previously^4^, we observed that SCI generated extensive infiltration of monocyte derived macrophages and activation of resident microglia (Fig. 1A,F,K). Similarly, we observed presumptive myelin debris, i.e. neutral lipid droplets detected by BODIPY staining inside TomL+ macrophages throughout the injury epicenter (Fig. 1B,G,L). Extracellular lipid droplets not clearly contained within macrophages were also present (Fig. 1B,G,L). Next, we sought to determine whether the cPLA_2_ system is active (phosphorylation indicates activation) in these chronically activated macrophages within the injured spinal cord. As indicated in Fig. 1C,H,M, cPLA_2_ is widely activated (p-cPLA_2_) throughout the lesion epicenter images of macrophages, and critically is observed within macrophages containing lipid debris at 28 days after injury (dpi). Similar observations were also seen at 7 dpi (Supplemental Fig. 1). Lastly, uninjured tissue from sham surgery animals contained TomL+ macrophage/microglia populations but no appreciable lipid debris, and minimal cPLA_2_ activation (Fig. 1P–T). Collectively this provides spatiotemporal evidence that macrophages are chronically present in the injured spinal cord, loaded with substantial myelin-derived lipid debris, and can contain activated cPLA_2._ Furthermore, these results provide the proof-of-concept that myelin and cPLA_2_ may play a key role in the prolonged pro-inflammatory macrophage activation thought to impede semi-acute and chronic recovery after SCI.

### Myelin potentiates pro-inflammatory macrophage activation in an activation state dependent manner

**Figure 2:**
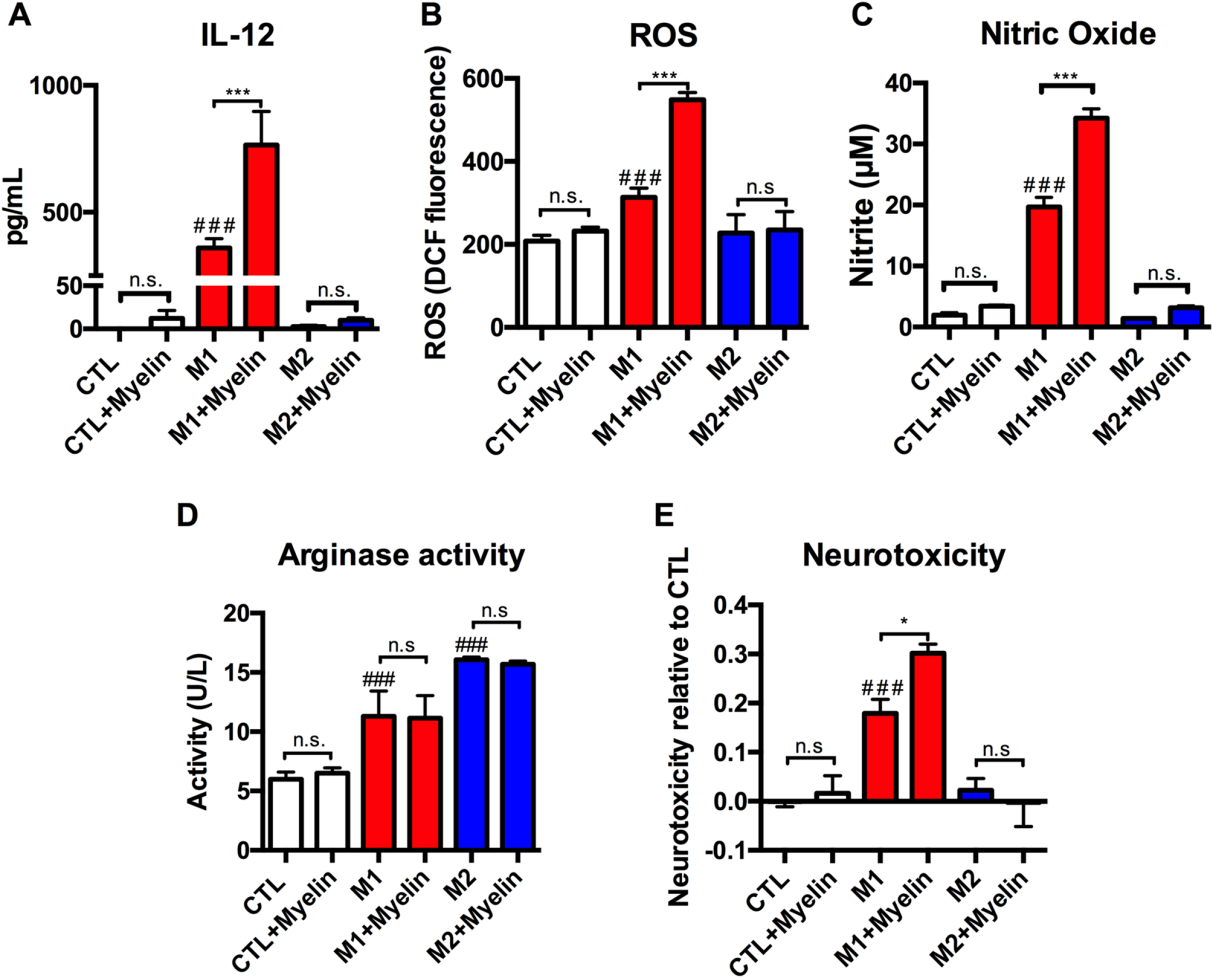
Myelin potentiates pro-inflammatory macrophage activation in vitro. Bone marrow derived macrophages (BMDMs) were utilized to determine the function outcomes resulting from myelin debris application under three distinct activation states: M1 (IFN-γ and LPS), M2 (IL-4), or CTL (media, i.e. unstimulated). (**A**) Myelin stimulation significantly increased pro-inflammatory IL-12 cytokine levels relative to M1 alone (***p < 0.001), however myelin did not increase IL-12 in CTL or M2 treated cells. M1 stimulations had increased IL-12 relative to CTL or M2 (^###^p < 0.001). (**B**) Following the stimulation BMDMs were washed and treated with 2 µM H2DCFDA for 25 min. Myelin stimulation increased ROS relative to M1 alone (***p < 0.001), however myelin did not increase ROS in CTL or M2 treated cells. M1 stimulations had increased ROS relative to CTL or M2 (^###^p < 0.001). (**C**) Nitric oxide levels in treated supernatants were examined using the Griess assay of nitrite accumulation. Myelin stimulation increased nitric oxide relative to M1 alone (***p < 0.001), however myelin did not increase nitric oxide in CTL or M2 treated cells. M1 stimulations had increased nitric oxide relative to CTL or M2 (^###^p < 0.001). (**D**) Cell lysates were tested for arginase enzymatic activity. M2 macrophages had higher arginase activity relative to M1 and CTL (p < 0.001 and p < 0.0001, respectively). The addition of myelin however did not significantly alter arginase activity in any of stimulations tested. (**E**) Supernatants were applied to a neuron culture (N2A cells) for 24 h to determine supernatant toxicity. Myelin stimulation increased neurotoxicity relative to M1 alone (*p < 0.05), however myelin did not increase neurotoxicity in CTL or M2 treated cells. M1 stimulations had increased ROS relative to CTL or M2 (^###^p < 0.001). Representative of 3 independent biological replications of both BMDMs and myelin source. *p < 0.05, **p < 0.01, ***p < 0.001, ^#^p < 0.05, ^##^p < 0.01, ^###^p < 0.001 mean ± SEM.

To determine the role of myelin in the activation of macrophages we developed an in vitro model of SCI inflammation. BMDMs are predictive of monocyte-derived macrophage responses in vivo in the injured spinal cord. This have been observed at transcription^26^ and functional levels^19^, as well as in response to therapeutic interventions^27^. Further, BMDMs are the primary myeloid cells phagocytizing myelin after SCI^4^. Specifically, we used an M1 stimuli (LPS + IFN-γ) to model the detrimental pro-inflammatory activation state, M2 (IL-4) to model the reparative anti-inflammatory activation state, and CTL (unstimulated control or M0) to model a mature, yet naïve macrophage. Myelin stimulation was generated from mouse CNS tissue to model the myelin debris “stimulant” generated after SCI in vivo. Using this system, we observe both myelin up-take across activation states and increased cPLA_2_ activation under M1 conditions (p < 0.001) (Supplementary Fig. 2). Interestingly, we observed that many of myelin’s effects on macrophage physiology were activation state dependent, with differential effects when applied in the M1, M2, or CTL activation states. In Fig. 2A, pro-inflammatory (M1) macrophages had increased production of the pro-inflammatory cytokine IL-12 compared to unstimulated (CTL) or anti-inflammatory (M2) macrophages, as would be expected (p < 0.001). Upon the concurrent addition of myelin to these groups, however, the M1 production of IL-12 rises substantially (p < 0.01), whereas CTL and M2 cells were not significantly affected. In Fig. 2B,C we performed additional stimulations and measured the production of reactive oxygen species (ROS) and nitric oxide, two toxic macrophage byproducts thought to contribute to cell death and SCI pathogenesis. With this, we observed a similar phenotype-specific effect in which only pro-inflammatory M1 cells were significantly potentiated by the addition of myelin, whereas CTL and M2 cells were not significantly affected. Specifically, M1 stimulation significantly increased levels of ROS (p < 0.001), and nitric oxide relative to unstimulated (CTL) cells, as would be expected. Critically, each of these M1 mediated increases were significantly increased with the application of myelin alongside the M1 stimulation, indicating that myelin potentiates pro-inflammatory responses (p < 0.001 and p < 0.001, respectively). Interestingly, this novel phenomenon occurred despite observing similar degrees of myelin debris uptake across all treatment groups (Supplemental Fig. 2), suggesting that differences in phagocytosis is not a contributing factor. The mechanisms through which the myelin was taken up under each phenotype, however, was not evaluated. To test the effects of myelin on an anti-inflammatory associated functional outcome we examined the activity of the arginase-1 enzyme in cell lysates from each of our stimulations (Fig. 2D). As would be expected, M2 macrophages had higher arginase activity relative to M1 and CTL (p < 0.001 and p < 0.0001, respectively). The addition of myelin, however, did not alter arginase activity in any of the stimulations tested. Lastly, the myelin stimulants did not contain any endotoxin contamination that would confound our results (Supplemental Fig. 3). Collectively, this suggests that the pro-inflammatory effects of myelin on macrophage activation states are phenotype-specific.

Next, we sought to determine the cellular implications of these shifts in macrophage polarization states by applying BMDM conditioned media from these cells to a neuronal cell line (N2A) to determine the relative neurotoxicity of each stimulation. In Fig. 2E, we observed that M1 neurotoxicity was again significantly potentiated by the addition of myelin, whereas CTL and M2 cell toxicity was unaffected by the addition of myelin.

### cPLA_2_ inhibition blocks myelin’s pro-inflammatory potentiation of M1 macrophages

**Figure 3:**
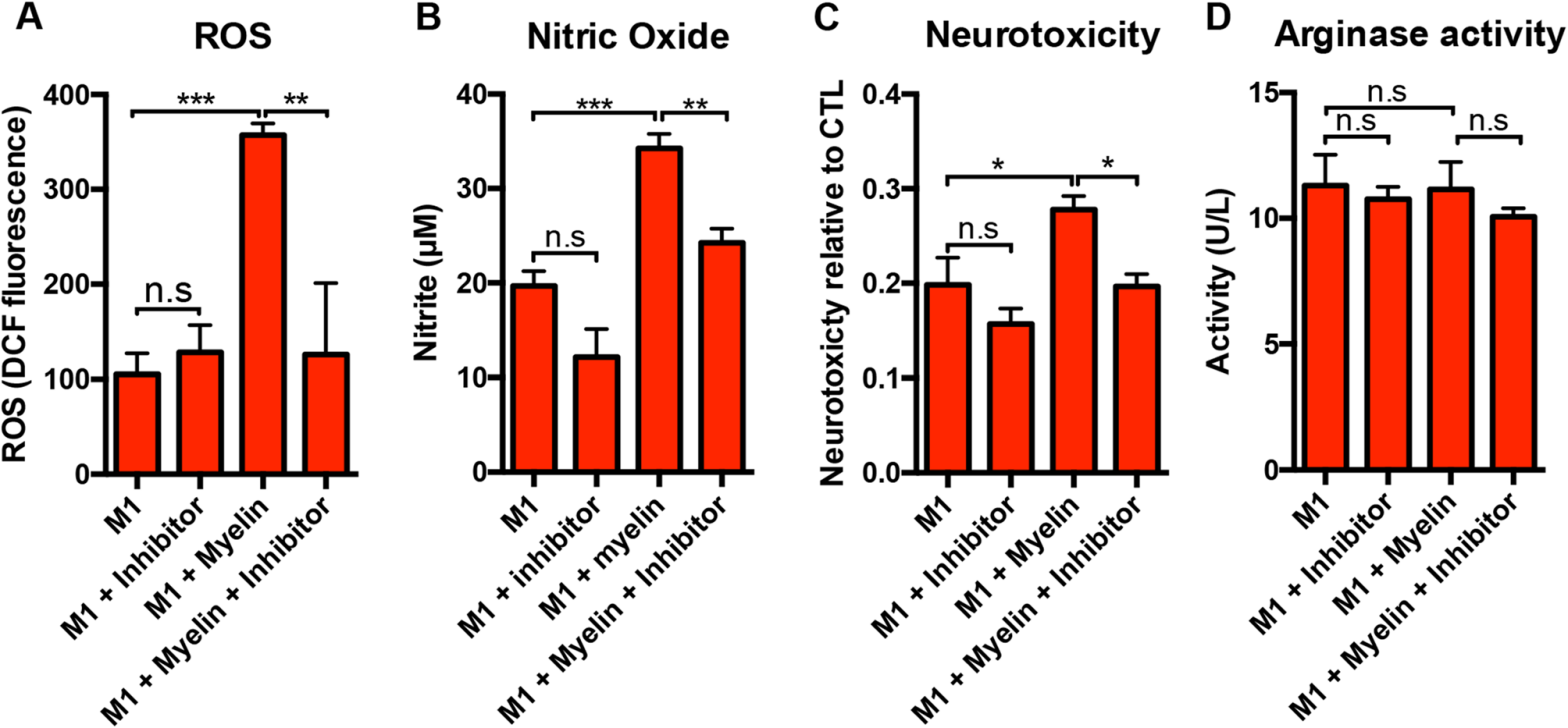
Inhibition of cPLA_2_ with PACOCF3 reduces the pro-inflammatory effects of myelin on M1 macrophages. BMDMs were grown and stimulated as in Fig. 2, with or without the addition of cPLA_2_ inhibitor, PACOCF3 (50 µM). (**A**) cPLA_2_ inhibition reduced myelin mediated ROS increases in M1 (IFN-γ and LPS) macrophages. (**B**) cPLA_2_ inhibition reduced myelin mediated nitric oxide production. (**C**) cPLA_2_ inhibition reduced the neurotoxic potential of M1 macrophages treated with myelin as determined by an MTT assay measurement of N2a cell viability. (**D**) cPLA_2_ inhibition did not significantly alter arginase activity. Representative of 3 biological replications of both BMDMs and myelin source. *p < 0.05, **p < 0.01, ***p < 0.001 mean ± SEM.

The activation of cPLA_2_ by inflammatory stimuli is thought of as the primary rate-limiting step in the release of AA and the initiation of the production of various eicosanoids during the onset of inflammation. Given that we observed significant effects of myelin in macrophages in the presence of pro-inflammatory stimuli (LPS and IFN-γ), we hypothesized that cPLA_2_ activation in M1 macrophages may be a key mediator of myelin’s cellular effects. To test this hypothesis, we targeted cPLA_2_ in our in vitro model using the chemical inhibitor Palmityl trifluoromethylketone (PACOCF3) as used previously^28^. As in our initial studies, we found myelin induced significant increases in ROS, nitric oxide, and neurotoxicity when applied with an M1 stimulus, potentiating the M1 pro-inflammatory response (Fig. 3). Critically, application of the cPLA_2_ inhibitor, PACOCF3, significantly reduced the myelin-mediated increases of ROS, nitric oxide, and neurotoxicity, indicating an important role for cPLA_2_ in this system (Fig. 3). Interestingly, PACOCF3 did not influence arginase activity in any of the stimulations tested (Fig. 3D and Supplemental Fig. 4D,H), suggesting that cPLA_2_ is not linked to cellular arginase activity. Given that myelin did not previously affect ROS, nitric oxide, neurotoxicity, or arginase in the context of CTL or M2 stimulation, we would not expect cPLA_2_ to influence these outcomes. Indeed, PACOCF3 had no significant effects on CTL or M2 simulations groups (Supplemental Fig. 4). Collectively these data suggest that cPLA_2_ may play a key role in mediating myelin’s effects on pro-inflammatory macrophage activation.

### Macrophage cytokine profiles indicate a mixed neuroinflammatory phenotype

**Figure 4:**
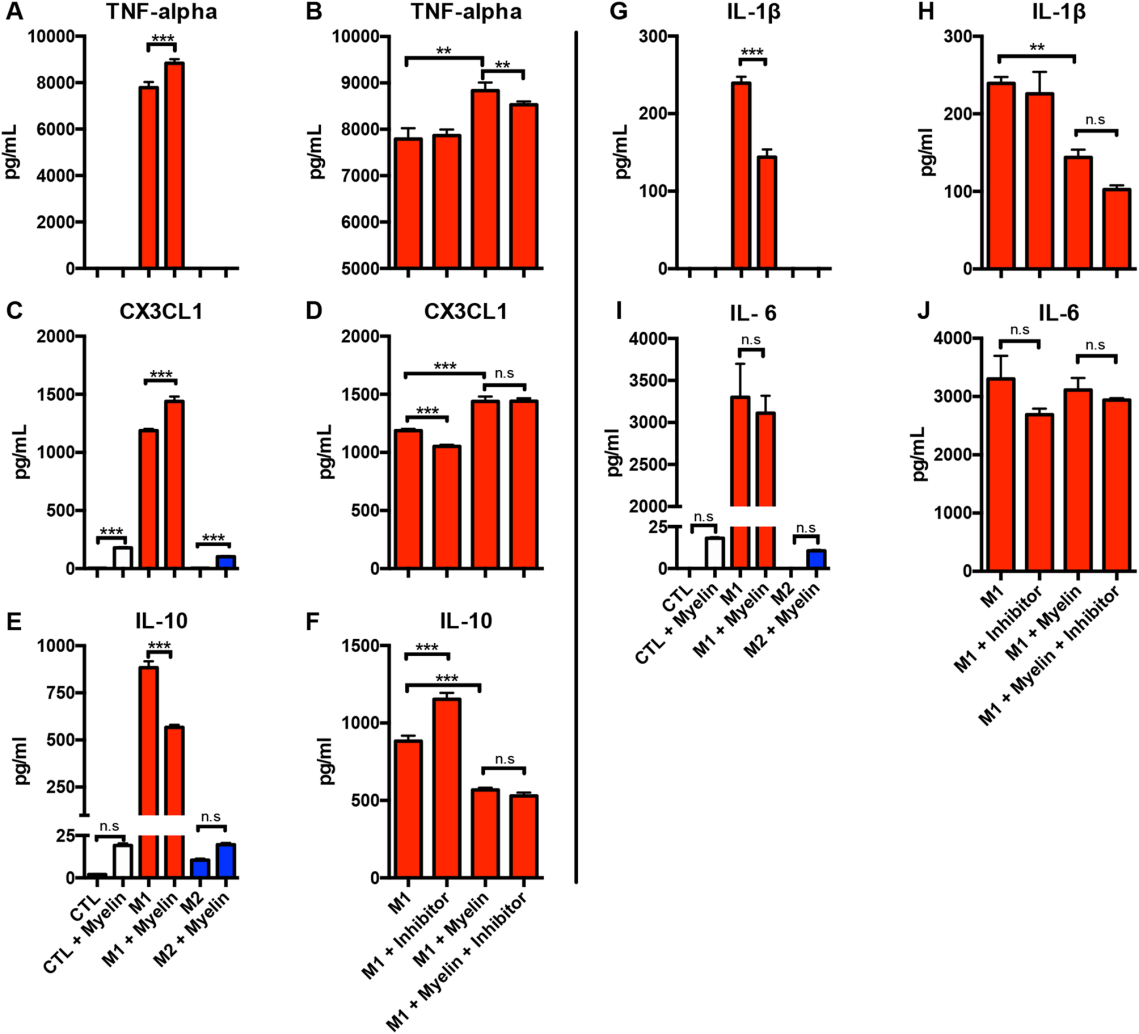
Macrophage cytokine profiles indicate a mixed neuroinflammatory phenotype. Supernatants from treated BMDMs were collected to measure pro- and anti-inflammatory cytokine production in response to phenotype, myelin stimulation, and cPLA_2_ inhibition with PACOCF3. (**A**, **B**) Myelin significanty increases the production of TNF-alpha in M1 cells, but has no effect on CTL and M2 cells. cPLA_2_ inhibition significantly reduced myelin potentiation of M1 TNF-alpha production. (**C**, **D**) Myelin increases CX3CL1 in CTL, M1, and M2 cells. cPLA_2_ inhibition significantly reduced this effect in M1 cells, but not under M1 and Myelin co-stimulation. (**E**, **F**) Myelin significantly decreased IL-10 production in M1 cells, but this effect was not reversed with cPLA_2_ inhibition. cPLA_2_ inhibition alone increased IL-10 production in M1 cells. (**G**, **H**) Myelin significantly reduced pro-inflammatory IL-1beta production in M1 cells, whereas CTL and M2 cells were unaffected. cPLA_2_ inhibition did not alter this effect. (**I**, **J**) Pro-inflammatory IL-6 was not significantly altered by myelin or cPLA_2_ inhibition. Refer to Supplementary Fig. 5 for CTL and M2 cPLA_2_ inhibition data. Representative of three biological replications. *p < 0.05 **p < 0.01, ***p < 0.001 mean ± SEM.

Macrophages exist along a spectrum of activation states commonly simplified to pro- vs. anti-inflammatory states in vivo, or M1/M2 in vitro. While these terms are used for practical purposes, macrophages are further characterized by many factors including morphology, surface markers, secreted cytokines, byproducts and functional outcomes. Our measures of neurotoxicity, IL-12, nitric oxide and ROS indicate a pathological phenotype; however, these are not comprehensive phenotypic indicators. To begin to address this complex phenotypic analysis we sought to measure additional inflammatory cytokines using a multiplex ELISA in our in vitro myelin/cPLA_2_ system. In line with our previous observations, M1 macrophages had significantly increased production of the pro-inflammatory cytokines TNFα (p > 0.001) and CX3CL1 (p > 0.001) upon the addition of myelin (Fig. 4A,C), and for TNFα, cPLA_2_ inhibition blunted this effect (p < 0.01) (Fig. 4B). Similarly, myelin significantly reduced the production of anti-inflammatory IL-10 in M1 cells, exacerbating their pro-inflammatory phenotype (p < 0.001) (Fig. 4E). Conversely, the pro-inflammatory cytokine IL-1β was significantly decreased in M1 cells following the addition of myelin (p < 0.001) (Fig. 4G). For the pro-inflammatory cytokine, IL-6, M1 cells were unaffected by the addition of myelin. Interestingly, for CX3CL1, myelin induced a small but significant increase in cytokine levels in CTL and M2 cells (Fig. 4C); IL-10 and IL-6 had similar trends in this regard, however, they were not statistically significant. Lastly, in some instances the cPLA_2_ inhibitor (PACOCF3) alone induces significant cytokine shifts, decreasing CX3CL1 (p < 0.01) and increasing anti-inflammatory IL-10 (p < 0.001) (Fig. 4D,F), suggesting that cPLA_2_ inhibition has anti-inflammatory effects outside the context of myelin stimulation. Interestingly, when examining the effects of myelin and cPLA_2_ inhibition specifically within CTL and M2 stimulation we observed statistically significant but biologically small, relative to M1, increases in both pro- (TNF-alpha, CX3CL1, IL-6) and anti-inflammatory (IL-10) cytokines suggesting myelin can induce a limited mixed phenotype in M2 and CTL groups (Supplemental Fig. 5). Collectively, these data indicate that myelin induces a mixed neuroinflammatory cytokine profile.

### Myelin increases cPLA_2_ activity in an activation state dependent manner

**Figure 5:**
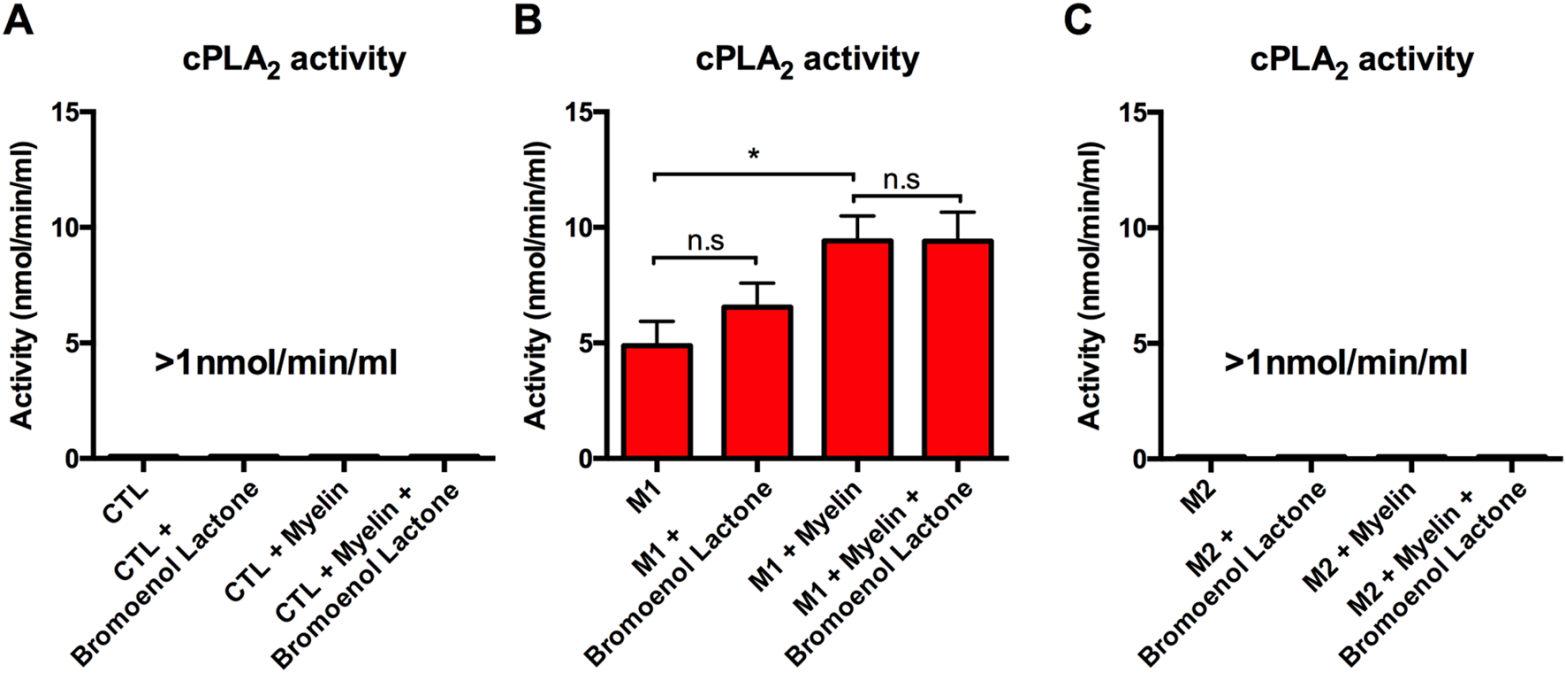
Myelin increases cPLA_2_ activity in an activation state dependent manner. Cell lysates were collected from BMDM cultures to measure cPLA_2_ activity following stimulation into CTL, M1, and M2 cells with or without myelin. Samples containing Bromoenol Lactone, an inhibitor of iPLA_2_ were used to exclude the possibility of iPLA_2_ contributing to enzymatic activity. (**A**) CTL treated cells had no discernable activity with or without myelin and Bromoenol Lactone. (**B**) M1 treated cells had a mean enzymatic activity of 4.88 nmol/min/mL. Addition of myelin significantly increased cPLA_2_ enzymatic activity (*p < 0.05). Addition of Bromoenol Lactone did not significantly alter enzyme activity. (**C**) M2 treated cells had no discernable activity with or without myelin and Bromoenol Lactone. Representative of three biological replications. *p < 0.05 **p < 0.01, ***p < 0.001 mean ± SEM.

Myelin has long been proposed as an inflammatory stimulus in CNS models of neuroinflammation^10^. Similarly, cPLA_2_’s activation in response to a wide variety of chemokines and inflammatory signal has been well documented^29^. Despite this, the formal hypothesis of whether myelin can directly increase macrophage cPLA_2_ activation has not been directly tested. To this aim we performed a PLA_2_ activity assay on cell lysates from CTL, M1, and M2 treated cells with or without myelin. Myelin increased PLA_2_ activity in an activation state dependent manner, with M1 cells containing active PLA_2_, which was further increased (*p < 0.05) with the addition of myelin (Fig. 5A–C). Conversely, PLA_2_ activity in CTL and M2 cells was below the detection limit of the assay with or without the addition of myelin. To distinguish cPLA_2_ activity from iPLA_2_, cell lysates were incubated in Bromoenol Lactone to inhibit iPLA_s._ iPLA_2_ inhibition did not significantly reduce total PLA_2_ activity in any of the groups tested indicating cPLA_2_ is likely the primary source of this enzymatic activity (Fig. 5A–C).

## Discussion

The continual inflammatory response observed after spinal cord injury (SCI) is a primary mechanism impairing recovery, however, the factors contributing to this maladaptive response are poorly understood. One unique aspect of the CNS injury environment not present in peripheral injuries is the accumulation of large volumes of myelin debris within phagocytes at the lesion epicenter. Indeed, recent literature suggests that myelin may drive pro-inflammatory macrophage activation, supporting the notion that myelin accumulation within macrophages may be a key driver of the persistent pro-inflammatory macrophage response observed after injury^4,10,30^. However, there are conflicting reports regarding the role of myelin on macrophage activation^7,31^ and the mechanisms governing these discrepancies are not well understood. In this study we provided the proof of concept that myelin and cPLA_2_ may play a key role in the prolonged pro-inflammatory macrophage activation thought to impede semi-acute and chronic recovery after SCI. Specifically, we found that myelin potentiates macrophage polarization in an activation state-dependent manner. The addition of myelin alongside a pro-inflammatory M1 macrophage stimulus (LPS and IFN-γ) further polarized pro-inflammatory activation as indicated by increased IL-12 production. Conversely, myelin had minimal effects on anti-inflammatory M2 (IL-4 stimulated) or control (unstimulated) macrophages. Similar patterns emerged when examining ROS, nitric oxide, arginase activity (indicative of M2 activation), and the neurotoxic potential of the macrophage supernatants. Inhibition of cPLA_2_ significantly blunted these harmful effects while arginase activity was unaffected. In vivo we observed ubiquitous myelin debris in, and around, macrophages expressing active cPLA_2_, providing the key spatiotemporal evidence that cPLA_2_ may also mediate the detrimental effects of myelin in macrophages after SCI.

Myelin and cPLA_2_’s pro-inflammatory activities appear to be largely exclusive to the M1 phenotype as M2 and CTL cells were unresponsive to myelin in most outcome measures. This interesting observation becomes clearer upon closer examination of cPLA_2_ biology. AA is stored at the sn-2 position of membrane phospholipids where it is largely inactive. Enzymes from the lipid-cleaving phospholipase A2 family can release AA from the membranes, of which cPLA_2_ is the most ubiquitous and widely studied due to its role in the targeted release of AA in response to a variety of agonists^29,32^. Here, we demonstrate that myelin itself can induce increased cPLA_2_ activity. Once activated, cPLA_2_ translocates from the cytosol to the membranes of the endoplasmic reticulum and nuclear envelope. These are the primary sites of cPLA_2_ activity under homeostatic conditions, however, any alterations in cPLA_2_ activity within the myelin-laden macrophage is currently unknown^33^. It remains uncertain if myelin’s AA rich lipids could be a direct substrate for cPLA_2_ or if these lipids could be trafficked within the cell to the endoplasmic reticulum and other membranes and be targeted. For each AA molecule released by cPLA_2_, lysophosphatidylcholine is also produced, which is known to cause further demyelination and thus could contribute to the prolonged myelin debris production observed after SCI^34^. Basal cPLA_2_ expression is increased in response to growth factors and inflammation in a variety of cell types, notably macrophages. cPLA_2_ is activated by mitogen-activated protein kinase (MAPK) phosphorylation of its serine 505 sites in response to increased intracellular calcium, inflammatory stimuli, or ROS, many of which are substantially elevated after SCI (or in our in vitro model LPS and IFN-γ)^14,35^. The amount of AA released by activated cPLA_2_ is then largely dependent on substrate availability, of which myelin could contribute substantial quantities to cellular stores in the membranes of the endoplasmic reticulum, nuclear envelope and other potential sites. Further, the oxidation of lipids in the membrane by ROS alters its viscosity further increasing AA availability to cPLA_2_^35^. Collectively, this suggests that the detrimental effects of myelin are restricted to the M1 phenotype, as only under these conditions could cPLA_2_ become robustly activated and release AA.

Given the influential roles cPLA_2_ activation and subsequent eicosanoid storms play in the initiation and resolution of inflammation, it is perhaps unsurprising that cPLA_2_ has been previously studied in the context of SCI. Indeed, following SCI there is a substantial acute and sustained production of AA derived inflammatory mediators^36–38^. One such study by NK Liu et al. found that pharmacologically or genetically targeting cPLA_2_ improved locomotor and anatomical recovery after SCI. Conversely, López-Vales et al. found cPLA_2_ to have protective functions after SCI. Given the immensely diverse factors controlling cPLA_2_ activation, AA release, and the downstream production of inflammatory mediators, it is quite possible that cPLA_2_ has differential effects depending on the treatment paradigm or cell type (i.e. toxic mediators produced by M1 macrophages as in our in vitro model, and anti-inflammatory mediators in other cell types with different enzymatic processing of AA released by cPLA_2_). Indeed, in the spinal cord after injury, in addition to macrophages, we observed appreciable cPLA_2_ activity in many non-macrophage cell types within the lesion epicenter. Given the great heterogeneity of lipid signaling molecules downstream of cPLA_2_, and differential capacities of each cell type to produce these mediators, it is uncertain whether cPLA_2_ is mediating detrimental or beneficial effects in these other non-macrophage cell types. Prior studies targeting cPLA_2_ have used a global approach to target cPLA_2_ either genetically or with chemical inhibitors with conflicting results^14,34^. Our results suggest that cPLA_2_ plays a clear detrimental role in macrophage physiological responses to myelin, and thus future studies specifically targeting macrophage cPLA_2_ in vivo may hold more therapeutic potential than the previous systemic approaches in which both detrimental and beneficial mechanisms are likely being affected. One important caveat to consider when using therapeutics targeting cPLA_2_ is that many chemical inhibitors are cross-reactive with closely related phospholipases. In this study our cPLA_2_ inhibitor of choice PACOCF3, for example, can also inhibit a related enzyme calcium-independent phospholipase A2 (iPLA_2_). While our data suggests that iPLA_2_ is not contributing significantly to total PLA_2_ activity at 24-h post-stimulation, it’s activities prior to cell lysis were not examined. Consequently, the influence of iPLA_2_ cannot be ruled out in this study. Similarly, previous work has demonstrated that inhibiting cPLA_2_ can influence the breakdown and phagocytosis of myelin during Wallerian degeneration^39^. Continued work is needed to determine if such a mechanism could be occurring in the SCI Myelin-macrophage and our in vitro model.

Our results are consistent with a number of previous observations. Specifically, a 1994 publication by Williams et al. demonstrated that the treatment of microglia with myelin debris increased microglial activation as indicated by increased pro-inflammatory cytokine and ROS production^10^. Next, Van der laan et al. demonstrated that myelin increases TNF-alpha and nitric oxide when applied to peritoneal macrophages. Lastly, Wang et al. (2014)^4^, observed similar increases in pro-inflammatory macrophage activation with myelin application to bone marrow-derived macrophages, and demonstrated the key role that this infiltrating macrophage population plays in clearing myelin debris in vivo after SCI.

While these papers support a mechanism linking myelin phagocytosis to the pro-inflammatory macrophage response observed after SCI, our results conflict with other data reporting anti-inflammatory actions of myelin in in vitro models of various CNS disorders^7,31^. This suggests that the effects myelin has on macrophage activation may depend on the type of macrophage, stimulation timing, myelin source, dosage, and the CNS condition being modeled as discussed previously^11^. Similarly, others have observed pro-inflammatory effects of myelin without a M1 co-stimulus^4^. Our data presented here indicate that the effect of myelin is phenotype specific, which may further account for these differences. For example, Kroner et al.,^7^ utilized a sequential approach: applying an M1 stimulus to the BMDMs, washed thoroughly, and then applied bovine-derived myelin. While this approach is certainly appropriate for some studies, it may not capture our phenotype-specific cPLA_2_ mediated inflammation, as cPLA_2_ could need sustained LPS/IFN-γ to remain activated and exert its effects with myelin. Specifically, cPLA_2_ activity is regulated by both a rapid transient calcium influx induced by inflammatory stimuli and its phosphorylation state allowing for complex regulation of either brief or sustained activation^40,41^. Given this, sequential or simultaneous application of myelin and LPS/IFN-γ could induce cPLA_2_ to activate and interact with myelin under very different regulatory conditions; however, further studies are needed to better understand how phenotype, cPLA_2_ regulation, and methodological variation such as this synergize to produce differential responses to myelin. In doing so we could better understand the immune dysregulation leading to chronic inflammation after human SCI.

Our model utilized in this work has its own strengths and limitations in modeling the complex SCI inflammatory response. First, we chose to use BMDM as our cell choice as recent literature has implicated this monocyte derived population as the primary mediator of myelin clearance, and as being more detrimental to recovery relative to microglia^4,42^. Second, we utilized a high dose of myelin which was not overtly toxic yet provided excess myelin to overwhelm the cell’s phagocytic capacity over the 24-h stimulation window similar to what occurs after SCI^4–6,9^. Lower doses may be better suited to shorter duration stimulations or studies investigating the effects of myelin upon binding to extracellular receptors. Third, our myelin preparations are from the same species and strain as the mice from which we collected the BMDMs (i.e. mouse myelin on mouse cells, and when feasible, myelin derived from the same mouse sacrificed for BMDM isolation). This was done to minimize any unintended cross-species immune effects. A limitation here, however, is that we used myelin derived from both brain and spinal cord tissue to yield sufficient myelin for our studies, it is possible that subtle differences in myelin composition between these sites could have differential effects. Similarly, the protein and lipid composition of CNS and PNS myelin differs and was not evaluated in this study^43^. Fourth, we utilized a prolonged 24-h stimulation paradigm to allow for complete lipid loading of the cells as occurs in vivo*.* It is certainly possible that the results could be different at earlier time points. Similarly, studies interested in true chronic effects could adapt these protocols using cell lines to overcome the short lifespan of BMDMs. Fifth, we applied the myelin and stimulants at the same time and for the entire stimulation, as this is what likely occurs after SCI. Lastly, we applied myelin with multiple types of stimulates to begin to capture how myelin may affect macrophages across the spectrum of phenotypic states. An important caveat to this, however, is that we primarily investigated pathological BMDM features associated with impaired SCI recovery. There are likely other effects of myelin on CTL and M2 macrophage physiology not captured in this study.

While in many ways our macrophage model closely replicates the cellular populations found after SCI^27^, there are certainly other factors not utilized here in our model. Notably, TNF and iron are implicated as key environmental mediators of detrimental macrophage activation^7^. Similarly, in vivo after SCI there are numerous other factors that can influence macrophage activation, including cross talk between macrophages and microglia, T-cell responses, and damage associated molecular patterns released by necrotic tissue^44,45^. While not investigated here, these are all likely important factors contributing to pro-inflammatory macrophage activation in myelin-laden macrophages and represent an important caveat in extrapolating our results to the human SCI condition. Similarly, while the data presented here implicate cPLA_2_ as an important regulator of myelin’s activity, other cellular mechanisms are certainly involved. Myelin uptake/phagocytosis, for example, is clearly a key step prior to any intracellular mechanism. Injection of myelin directly into the spinal cord previously by Sun and colleagues was found to induce leukocyte chemoattraction, however, this effect was lost in CR3 KO animals with deficient phagocytic capacity. Next, they implicated the FAK/Akt/NF-κB signaling cascade as a mediator of myelin’s activity^46^. These observations are certainly compatible with our current observations as the NF-κB signaling cascade is activated by similar stimuli as the cPLA_2_ signaling cascade including our LPS in-vitro stimulant. Interestingly, activation of NF-κB by myelin may even drive increased cPLA_2_ expression, further demonstrating the intertwined nature of many proposed mechanisms^47^. Collectively, this demonstrates the need for continued investigation of these pathways to identify a clinically viable treatment for SCI in the human condition.

Our in vitro data suggest that myelin debris potentiates pro-inflammatory functions specifically within the M1 macrophage population. Intriguingly, the time course of peripheral macrophage infiltration and initial clearance of myelin debris between 3 and 7 days post-SCI correlates with the peak presence of M2 macrophage activation markers, which then progressively drop as the cells shift to a pronounced and prolonged M1 activation state^1,2^. By chronic time points only M1 markers can be detected, indicative of the prolonged pro-inflammatory macrophage activation thought to impede recovery^4,6,9^. Here we demonstrate that cPLA_2_ is present within myelin-laden macrophages well into chronic time points. Together with our in vitro data, this provides the proof of concept that myelin and cPLA_2_ may play a key role in the prolonged pro-inflammatory macrophage activation thought to impede semi-acute and chronic recovery after SCI. Collectively this highlights macrophage cPLA_2_ activity as a potential key mediator of the neuroinflammatory response after SCI and thereby warrants continued investigation as a therapeutic target.

## Conclusions

Spinal cord injury (SCI) produces chronic intraspinal inflammation consisting of resident microglia and infiltrating monocytes. These chronically activated SCI macrophages adopt a persistent pro-inflammatory, pathological state that potentiates secondary damage and impairs SCI recovery^1,3^. The mechanisms driving chronic macrophage activation in SCI are poorly understood. Here we implicate myelin debris and cPLA_2_ as key mediators of pathological macrophage activation. In vitro we found that the effects of myelin on macrophage activation are phenotype-specific, with myelin potentiating only pro-inflammatory (LPS + INF-γ; M1) macrophage activation, whereas myelin had no pro-inflammatory effect on unstimulated or anti-inflammatory (IL-4; M2) macrophages. Inhibition of cPLA_2_ significantly reduced the detrimental effects of myelin on M1 macrophages, implicating cPLA_2_ as a key regulator of maladaptive macrophage activation. In vivo we observed ubiquitous myelin debris in, and around, macrophages expressing active cPLA_2_ providing the key spatiotemporal evidence that cPLA_2_ may also mediate the detrimental effects of myelin in macrophages after SCI. Collectively, this establishes a novel mechanism driving detrimental macrophage activation and provides key evidence identifying macrophage cPLA_2_ activity as a therapeutic target to improve recovery after SCI.

## Supplementary Information

Supplementary Legends.

Supplementary Figure 1.

Supplementary Figure 2.

Supplementary Figure 3.

Supplementary Figure 4.

Supplementary Figure 5.

## Data Availability

The datasets analyzed during the current study are available from the corresponding author upon request. In vivo data will be made available through the Open Data Commons for Spinal Cord Injury (ODC-SCI: https://scicrunch.org/odc-sci).

## Abbreviations

AA: Arachidonic acid
BMDM: Bone marrow-derived macrophage
cPLA_2_: Cytosolic phospholipase A2
p-cPLA_2_: Phosphorylated cytosolic phospholipase A2 (activated)
IFN: Interferon
IL: Interleukin
LPS: Lipopolysaccharide
MCM: Macrophage conditioned media
P/S: Penicillin/streptomycin
ROS: Reactive oxygen species
RPMI: Roswell Park Memorial Institute medium
SCI: Spinal cord injury
