# Supplementary material for: The effects of myelin on macrophage activation are phenotypic specific via cPLA2 in the context of spinal cord injury inflammation
Source: Sci Rep. 2021 Mar 18;11:6341. doi: 10.1038/s41598-021-85863-6 (PMC7973514; doi:10.1038/s41598-021-85863-6)
Supplement: Supplementary file 1 — Supplementary Legends. [file 41598_2021_85863_MOESM1_ESM.docx]

**The Effects of Myelin on Macrophage Activation are Phenotypic Specific via cPLA_2_ in the Context of Spinal Cord Injury Inflammation**

Timothy J. Kopper1, Bei Zhang1, William M. Bailey1, Kara E. Bethel1, John C. Gensel1,*

1 Spinal Cord and Brain Injury Research Center, Department of Physiology, University of Kentucky College of Medicine, Lexington, KY, 40536, USA

* Correspondence:
John C. Gensel, PhD Email: gensel.1@uky.edu

**Figure Legends:**

**Supplemental Figure 1. Macrophages can contain both myelin-derived lipids and active cPLA_2_ 7 days after spinal cord injury (SCI).** Eight adult, 4-month-old C57/b female mice received a T9 75kdyn infinite horizons (IH) contusion SCI. A-E) Representative examples of TomL positive macrophages (blue) in injured white matter containing both lipid debris (green, BODIPY, staining for neutral lipids) and active p-cPLA_2_ (red). DAPI (white) was excluded from merged image. Imaged area is represented by box within spinal cord diagram. Boxes on image indicate examples of triple positive cells. Maximum intensity projection confocal images. Scale bar in image A=25µm

**Supplemental Figure 2. *In Vitro* modeling of cPLA_2_ activity in myelin loaded macrophages.**

Coverslip plated BMDMs were stained for TomL (macrophage marker, blue), BODIPY (myelin derived lipids, green) and active p-cPLA_2_ (red). Myelin uptake was quantified as the threshold area ratio of BODIPY or p-cPLA_2_ to TomL positive stain. A-F) **Representative images of CTL, M1, and M2** BMDMs treated with or without myelin debris. G) CTL, M1, and M2 BMDMs treated with myelin contained significantly more neutral lipids (BODIPY) than each of their respective untreated counterpart (### p<0.001); however, there were no significant differences between groups treated with myelin. H) M1 stimulated BMDMS has significantly higher p-cPLA_2_ immunoreactivity than either M1 or M2 (### p<0.001); however, myelin was not found to alter p-cPLA_2_ immunoreactivity in any of the groups tested.

**Supplemental Figure 3. Myelin stimulants did not contain detectable endotoxin contamination.** Aliquots from each myelin isolation were stored at -80 °C prior to testing. Myelin stimulants and negative control (1x sterile PBS) had endotoxin levels below the level of detection (<.055 EU/mL). The positive control (LPS 50 ng/mL) exceeded the detection limit (>1.045 EU/mL). mean ± SEM.

**Supplemental Figure 4. cPLA_2_ inhibition has no effects on CTL or M2 stimulated cells.** Cells were grown and stimulated as detailed in Figures 2 and 3. A-D) cPLA_2_ inhibition did not significantly change levels of reactive oxygen species (ROS), nitric oxide, supernatant neurotoxicity, or arginase activity in CTL (unstimulated) cells with or without the addition of myelin. E-H) cPLA_2_ inhibition did not significantly change levels of reactive oxygen species (ROS), nitric oxide, supernatant neurotoxicity, or arginase activity in M2 (IL-4) cells with or without the addition of myelin. Representative of 3 biological replications of both BMDMs and myelin source n.s.=non-significant, p>0.05, mean ± SEM.

**Supplemental Figure 5.** **Macrophage cytokine profiles indicate a mixed neuroinflammatory phenotype in CTL and M2 stimulated cells in response to myelin and inhibition of cPLA_2_ with PACOCF3.** Supernatants from treated BMDMs were collected to measure pro and anti-inflammatory cytokine production in response to phenotype, myelin stimulation, and cPLA_2_ inhibition with PACOCF3. A-J) Myelin significantly increases the production of pro and anti-inflammatory cytokines TNF-alpha, CX3CL1, IL-10 and IL-6. IL-1Beta was unaffected. cPLA_2_ inhibition induced small but statistically significant adjustments in cytokine levels in myelin treated CTL and M2 cells. Representative of 3 biological replications. *p<0.05 ** p<0.01, *** p<0.001 mean ± SEM.
