# Supplementary figures and images for: The effects of myelin on macrophage activation are phenotypic specific via cPLA2 in the context of spinal cord injury inflammation
Source: Sci Rep. 2021 Mar 18;11:6341. doi: 10.1038/s41598-021-85863-6 (PMC7973514; doi:10.1038/s41598-021-85863-6)

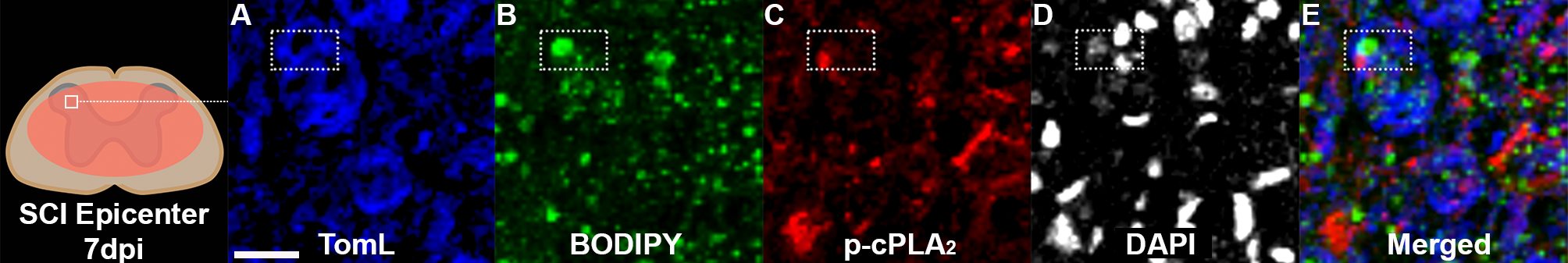

Supplement: Supplementary file 2 — Supplementary Figure 1. [file 41598_2021_85863_MOESM2_ESM.tif]

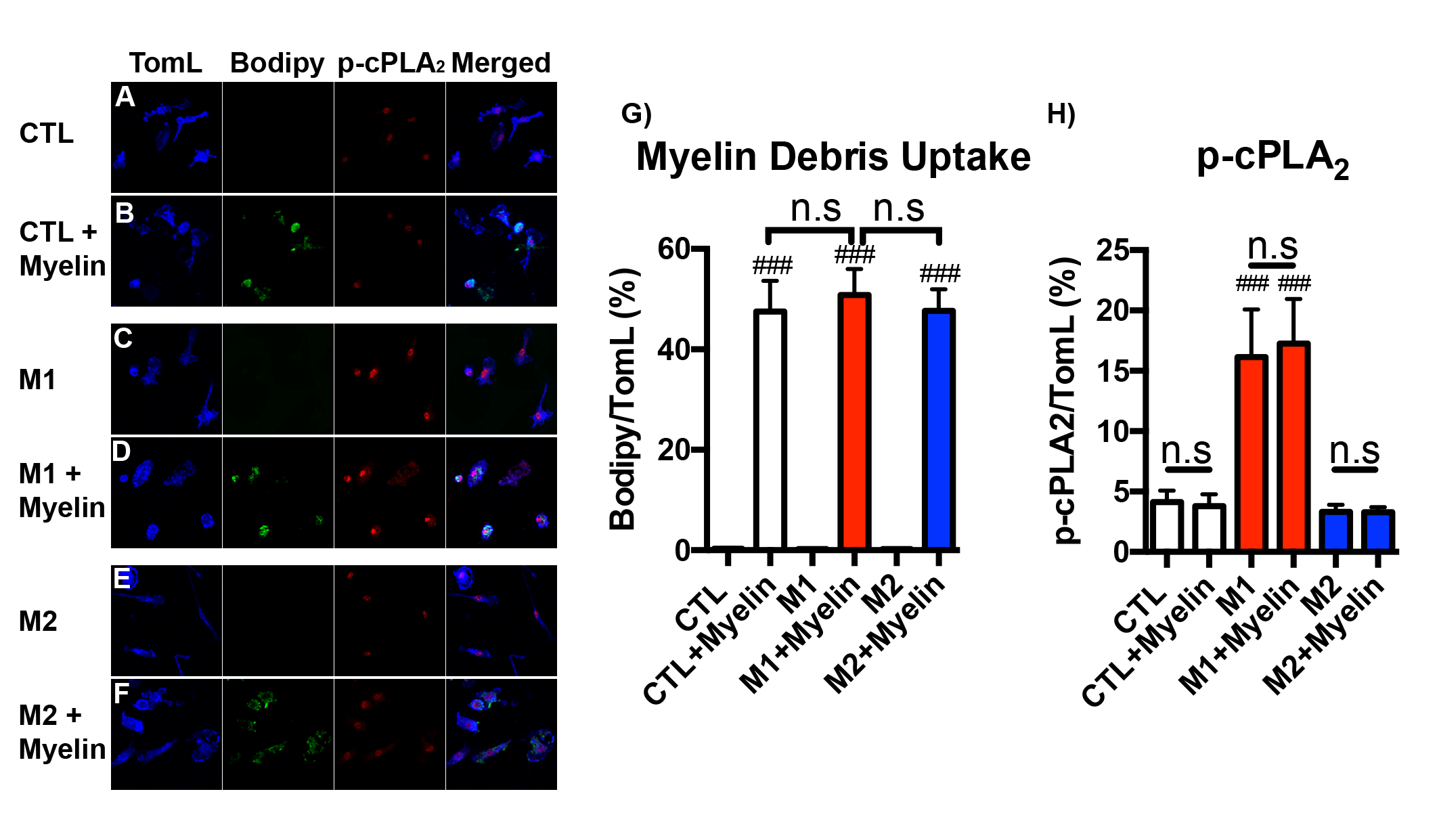

Supplement: Supplementary file 3 — Supplementary Figure 2. [file 41598_2021_85863_MOESM3_ESM.tif]

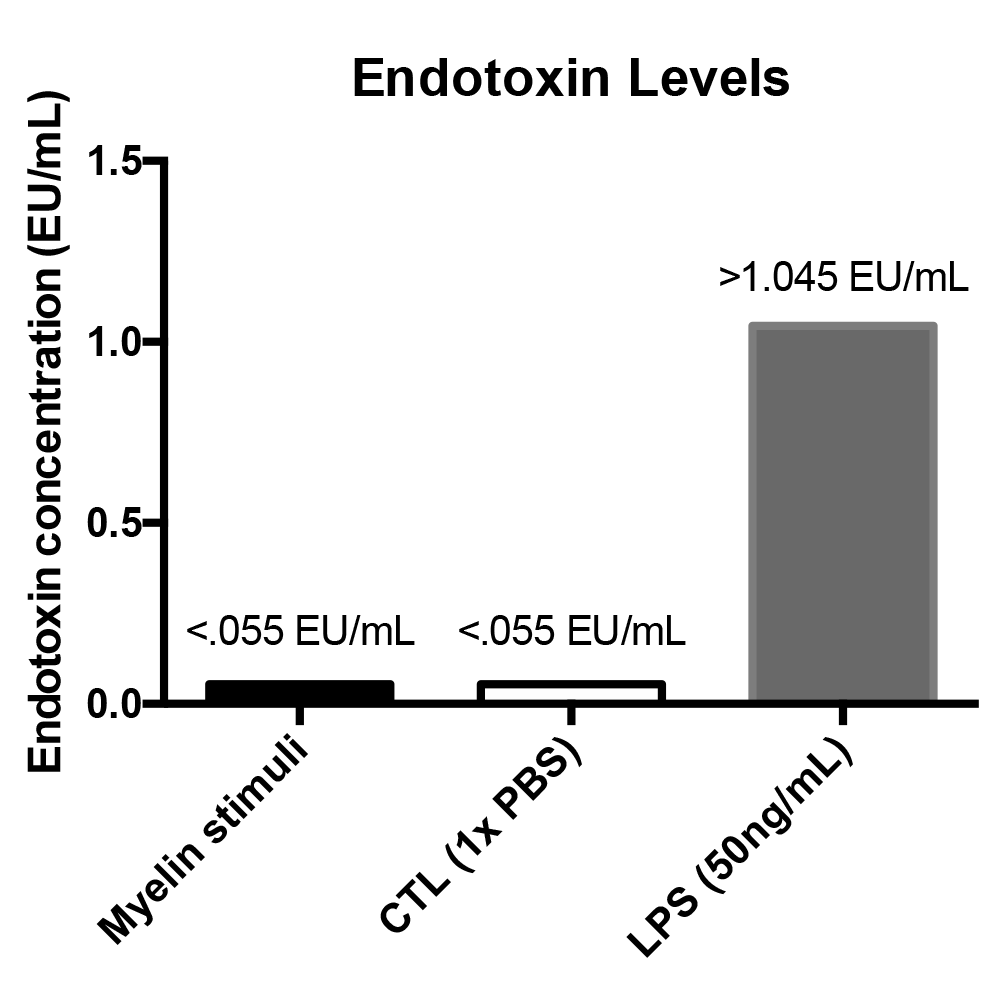

Supplement: Supplementary file 4 — Supplementary Figure 3. [file 41598_2021_85863_MOESM4_ESM.tif]

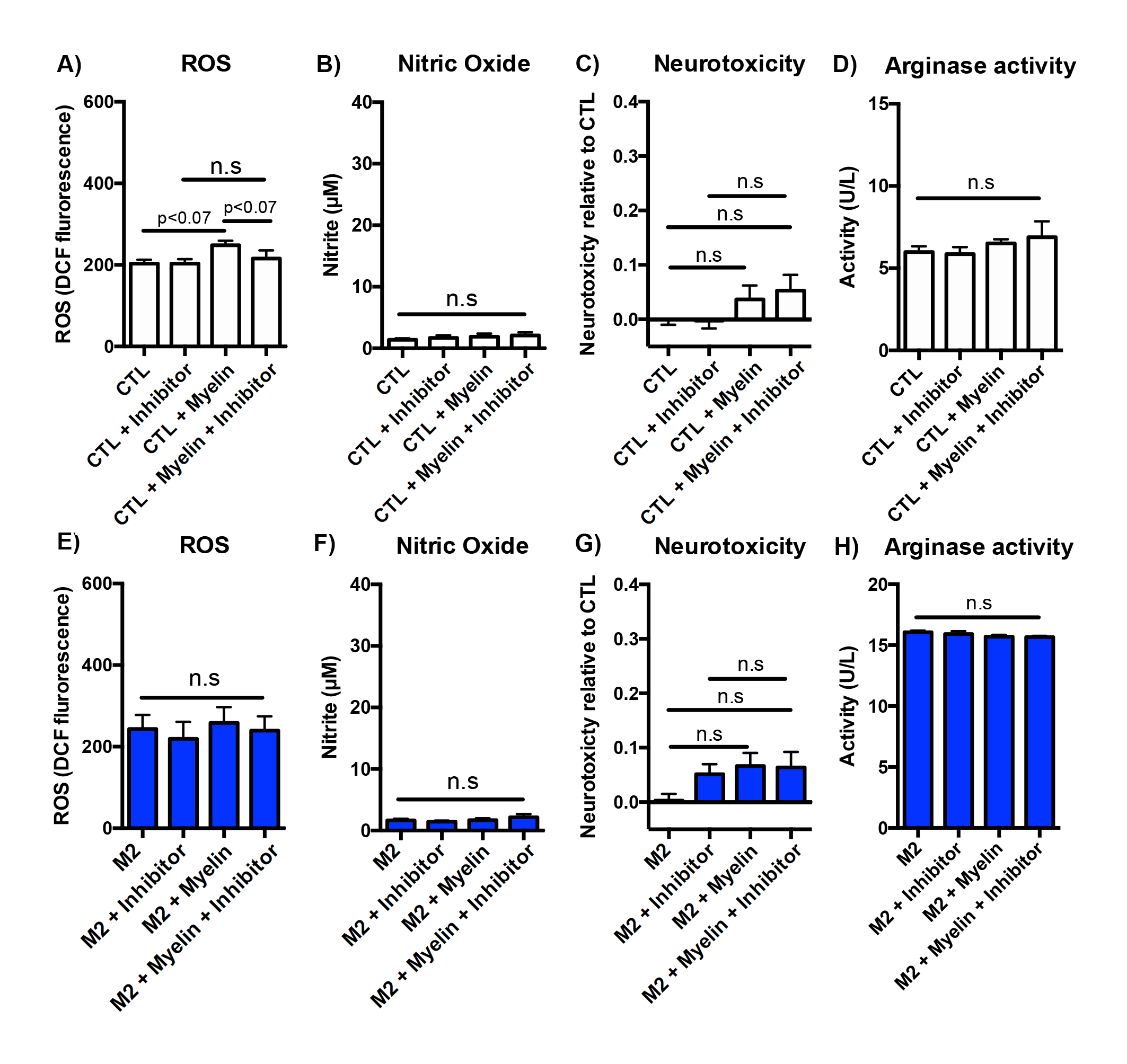

Supplement: Supplementary file 5 — Supplementary Figure 4. [file 41598_2021_85863_MOESM5_ESM.tif]

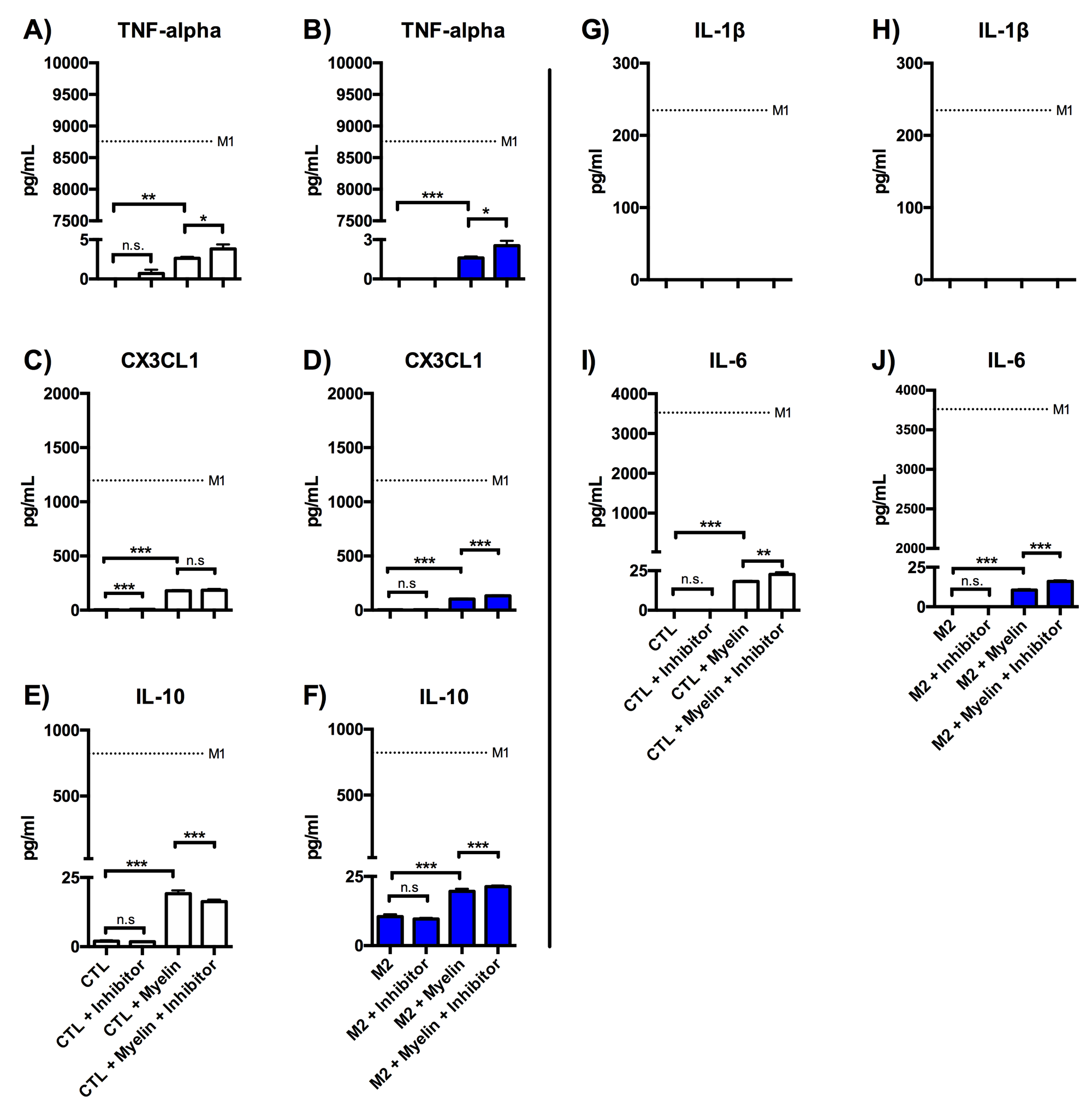

Supplement: Supplementary file 6 — Supplementary Figure 5. [file 41598_2021_85863_MOESM6_ESM.tif]
